# Potent *in vitro* synergistic antiviral effects of the pan-coronavirus fusion inhibitor EK1 in combination with RBD-specific antibodies or M^pro^ inhibitors

**DOI:** 10.1128/jvi.00076-26

**Published:** 2026-03-30

**Authors:** Ruixue Xiu, Yuanzhou Wang, Wenbo Cai, Qian Wang, Minxiang Xie, Yingdan Wang, Cheng Li, Qiao Wang, Jinghe Huang, Tianlei Ying, Chuanjun Song, Lu Lu, Shibo Jiang, Wei Xu

**Affiliations:** 1 Key Laboratory of Medical Molecular Virology (MOE/NHC/CAMS), Shanghai Institute of Infectious Disease and Biosecurity, Shanghai Frontiers Science Center of Pathogenic Microorganisms and Infection, School of Basic Medical Sciences, Shanghai Public Health Clinical Center, Shanghai Medical College, Fudan University 12478, Shanghai, China; 2 College of Chemistry, Pingyuan Laboratory, Zhengzhou University 12636, Zhengzhou, Henan, China; University of North Carolina at Chapel Hill, Chapel Hill, North Carolina, USA

**Keywords:** pan-coronavirus fusion inhibitor, main protease inhibitor, coronavirus, trident antiviral therapy

## Abstract

The continuous evolution of coronaviruses compromises the efficacy of existing therapeutic antibodies and replication inhibitors, necessitating antiviral strategies that are resilient to viral escape. Distinct from conventional antibody cocktails that remain vulnerable to spike protein mutations, we evaluated a multi-mechanistic therapy targeting three discrete stages of the viral life cycle: receptor binding, membrane fusion, and viral replication. Using pseudotyped and authentic human coronaviruses (HCoVs), including SARS-CoV-2 and HCoV-OC43, in cell culture models, we demonstrated that combining the pan-coronavirus fusion inhibitor EK1 with a receptor-binding domain-specific broadly neutralizing antibody (bnAb) and/or a main protease (M^pro^) inhibitor yields synergistic inhibition. Collectively, our findings establish a conceptual framework for a trident antiviral regimen that theoretically restricts the evolutionary space for viral escape, although clinical translation will require optimizing delivery strategies to reconcile the distinct pharmacokinetic profiles of these agents.

**IMPORTANCE:**

The continuous emergence of highly transmissible and immune-evasive SARS-CoV-2 variants highlights an urgent need for broad-spectrum antiviral approaches capable of countering rapid viral evolution and future coronavirus outbreaks. Here, we designed a dual- or triple-stage antiviral strategy centered on the membrane fusion inhibitor EK1, in combination with a receptor-binding domain-targeting antibody and/or a small-molecule viral replication inhibitor (M^pro^ inhibitor). In cell culture models, our results demonstrate marked synergy between EK1 and the antibody and/or the replication inhibitor. These combinations not only enhance antiviral potency but are also anticipated to maintain efficacy against emerging variants. By targeting non-overlapping stages of the viral life cycle, this approach theoretically raises the genetic barrier to resistance. While clinical translation will require reconciling the distinct pharmacokinetic profiles of these components, our study provides a conceptual framework for developing a trident anti-coronavirus therapy.

## INTRODUCTION

Coronaviruses pose a persistent and evolving threat to global public health. The 21st century is marked by three major zoonotic outbreaks: severe acute respiratory syndrome coronavirus (SARS-CoV) in 2002/2003, Middle East respiratory syndrome coronavirus (MERS-CoV) in 2012, and severe acute respiratory syndrome coronavirus 2 (SARS-CoV-2) since 2019. These viruses have caused severe respiratory illnesses, widespread mortality, and profound socioeconomic disruption (1–5).

**Fig 1:**
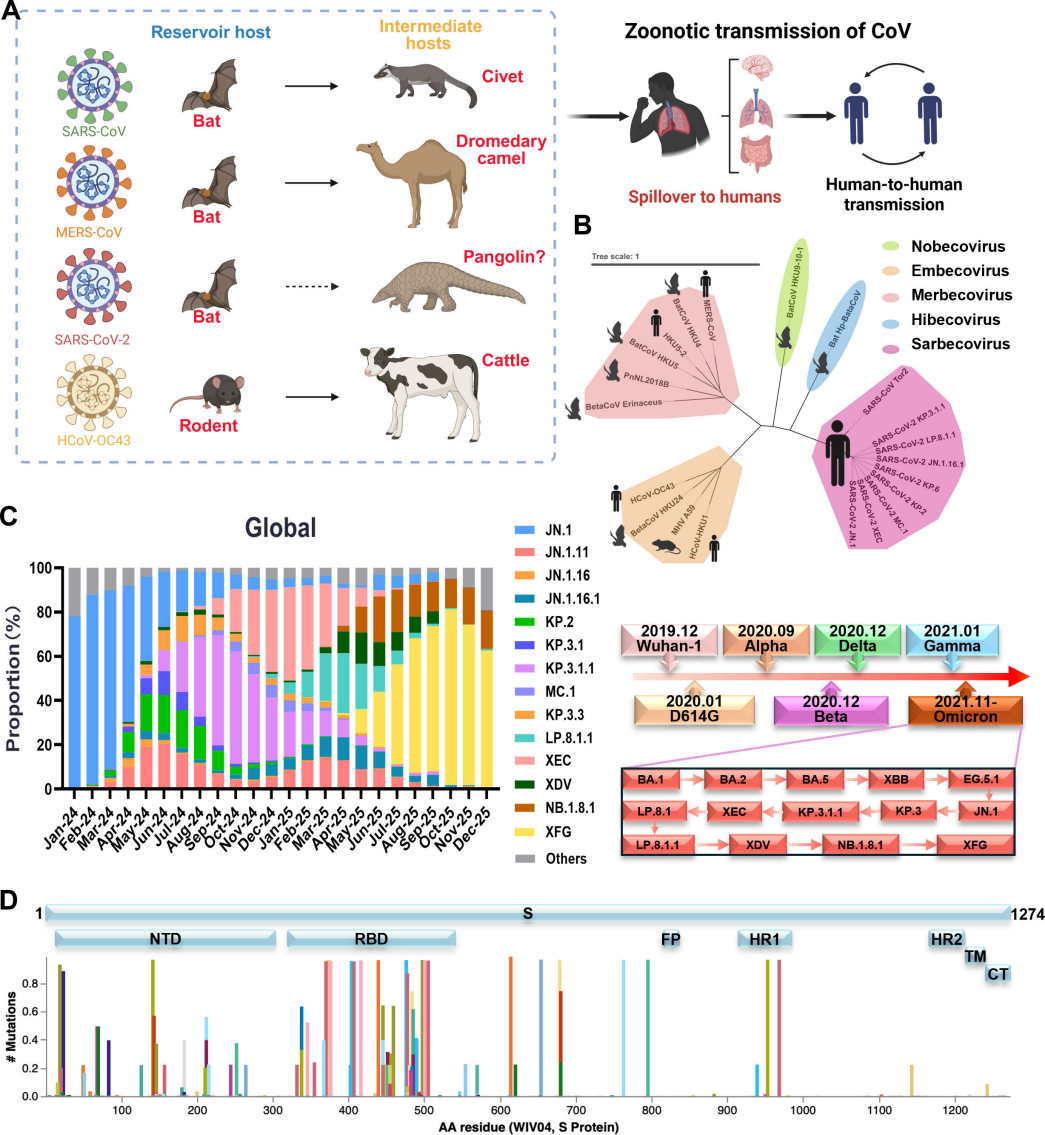
Overview of SARS-CoV-2 evolution and diversity. (**A**) Coronaviruses are capable of infecting a wide range of mammals, including bats, civets, dromedary camels, and humans, thus posing a potential risk of cross-species transmission. (**B**) Phylogenetic tree of β-CoV classified into five distinct subgenera. (**C**) Global frequencies of circulating SARS-CoV-2 variants, including JN.1, JN.1.11, JN.1.16, JN.1.16.1, KP.2, KP.3.1, KP.3.1.1, LP.8.1.1, MC.1, KP.3.3, XEC, XDV, NB.1.8.1, and XFG from January 2024 to December 2025. Data were obtained from covSPECTRUM (10). (**D**) Based on 93,925 SARS-CoV-2 S protein sequences from China collected via the GISAID database from November 2019 to December 2025, this figure illustrates the distribution of mutations across the S protein (amino acid residues 1–1,274, relative to the WIV04 reference genome). All sequences used are ≥29,000 bases in length and contain no more than 5% ambiguous bases. Data are sourced from the COVID-19 CG database (https://covidcg.org).

Recent metagenomic surveys of wildlife further highlight the latent risk of future spillovers: diverse betacoronaviruses (β-CoVs) in bats and pangolins form complex transmission chains through interactions with livestock and captive animals, while phylogenetic classification of β-CoVs into five subgenera (Sarbecovirus, Merbecovirus, Embecovirus, Hibecovirus, Nobecovirus) underscores their genetic diversity and potential for cross-species adaptation (Fig. 1A and B) (6–9).

SARS-CoV-2, the causative agent of COVID-19 (11), exemplifies the challenges of viral evolution. While SARS-CoV-2 has undergone continuous and rapid diversification since the emergence of the Alpha and Omicron variants, the recent evolutionary landscape in 2024–2025 has been characterized by the rise of highly immune-evasive lineages such as XFG and NB.1.8.1 (Fig. 1C) (10, 12–17). Analysis of 93,925 SARS-CoV-2 S protein sequences from China (https://covidcg.org) reveals that mutations are disproportionately concentrated in the receptor-binding domain (RBD) and N-terminal domain (NTD), which are key regions targeted by neutralizing antibodies, directly undermining the efficacy of antibody-based therapies (Fig. 1D) (11, 18, 19). The resistance-associated mutations in the main protease (M^pro^) have also reduced the long-term efficacy of M^pro^ inhibitors such as Paxlovid, highlighting a critical limitation of single-target therapeutics in the face of ongoing viral evolution (20).

Currently, COVID-19 interventions primarily rely on single-stage or single-target strategies: vaccines and neutralizing antibodies target the RBD to block viral entry (21–24), while small-molecule drugs like remdesivir and Paxlovid inhibit viral replication (25, 26). Although cocktail therapies—combining non-competing antibodies or antibodies with antiviral drugs—have shown promise in reducing resistance risk, they rarely address both viral entry and replication stages simultaneously (27–29). A notable exception is the fusion inhibitor EK1, which can competitively bind to heptad repeat 1 in the spike protein, blocking the formation of the six-helix bundle (6-HB) between viral HR1 and HR2 regions required for viral-cellular membrane fusion (30–34). The strategy of combining an entry inhibitor with a neutralizing antibody has been previously validated by our group, demonstrating significant synergy against MERS-CoV infection by simultaneously blocking viral attachment and membrane fusion (35).

Distinct from previous combinatorial strategies that primarily rely on antibody cocktails or antibody-polymerase inhibitor pairs, this study represents a systematic evaluation of a “triple-blockade” strategy targeting the viral life cycle at three spatiotemporally distinct steps: receptor binding, membrane fusion, and proteolytic processing. A key innovation of our approach is the incorporation of the pan-coronavirus fusion inhibitor EK1 as a conserved backbone. As described in our previous publications (36, 37), although the HR1 regions in the S2 proteins of different human coronaviruses (HCoVs) do exhibit some sequence variability, they are relatively conserved. Crucially, the residues at *a, d, e,* and *g* positions of the HR1 helical wheel and those at the *a* and *d* positions of the HR2 helical wheel are highly conserved due to their hydrophobic nature. Alterations at these sites would disrupt trimerization of HR1 or the formation of the six-helix bundle (6-HB) between HR1 and HR2. In contrast, the residues at the *b, c,* and *f* positions of HR1 helical wheel (generally hydrophilic) are tolerant to variation. Accordingly, the hydrophobic residues at the *a* and *d* positions of EK1 interact with the conserved *e* and *g* residues of HR1 across diverse HCoVs, enabling 6-HB formation and thereby inhibiting viral fusion with host cell membranes. This conserved interaction underpins EK1’s broad-spectrum activity. We hypothesized that this multi-mechanistic combination would not only yield potent synergistic antiviral effects, allowing for significant dose reductions, but also create a high genetic barrier capable of countering immune-evasive variants (e.g., XFG and NB.1.8.1) that render monotherapies ineffective.

To overcome the limitations of single-target therapies and leverage the conserved nature of HR1, we developed a rational combination therapy strategy that simultaneously targets distinct stages of the viral life cycle: receptor-binding (via RBD-specific broad-neutralizing antibodies [bnAbs]), fusion (via EK1), and replication (via M^pro^ inhibitors). This multi-stage approach aims to exploit complementary mechanisms of action: bnAbs provide targeted inhibition of viral attachment to host cells, EK1 blocks virus-cell membrane fusion independently of RBD mutations, and M^pro^ inhibitors disrupt viral replication.

In this study, we evaluated the efficacy of a combination strategy using pseudotyped and authentic coronaviruses, including SARS-CoV-2 variants (e.g., KP.3.1.1) and HCoV-OC43 (an Embecovirus). We first tested dual combinations (EK1 + bnAb or EK1 + M^pro^ inhibitor) to assess synergistic antiviral effects. We then investigated the triple combination (EK1 + bnAb + M^pro^ inhibitor) to determine if it further enhances efficacy. Our results demonstrate that multi-stage intervention not only exhibits potent antiviral activity across diverse coronaviruses but also provides a conceptual framework and *in vitro* proof-of-concept readily adaptable template for next-generation therapeutics—critical for addressing both current SARS-CoV-2 variants and future emerging coronavirus infections.

## RESULTS

### EK1 improves neutralization potency of broadly neutralizing antibodies against pseudotyped and authentic SARS-CoV-2

**Fig 2:**
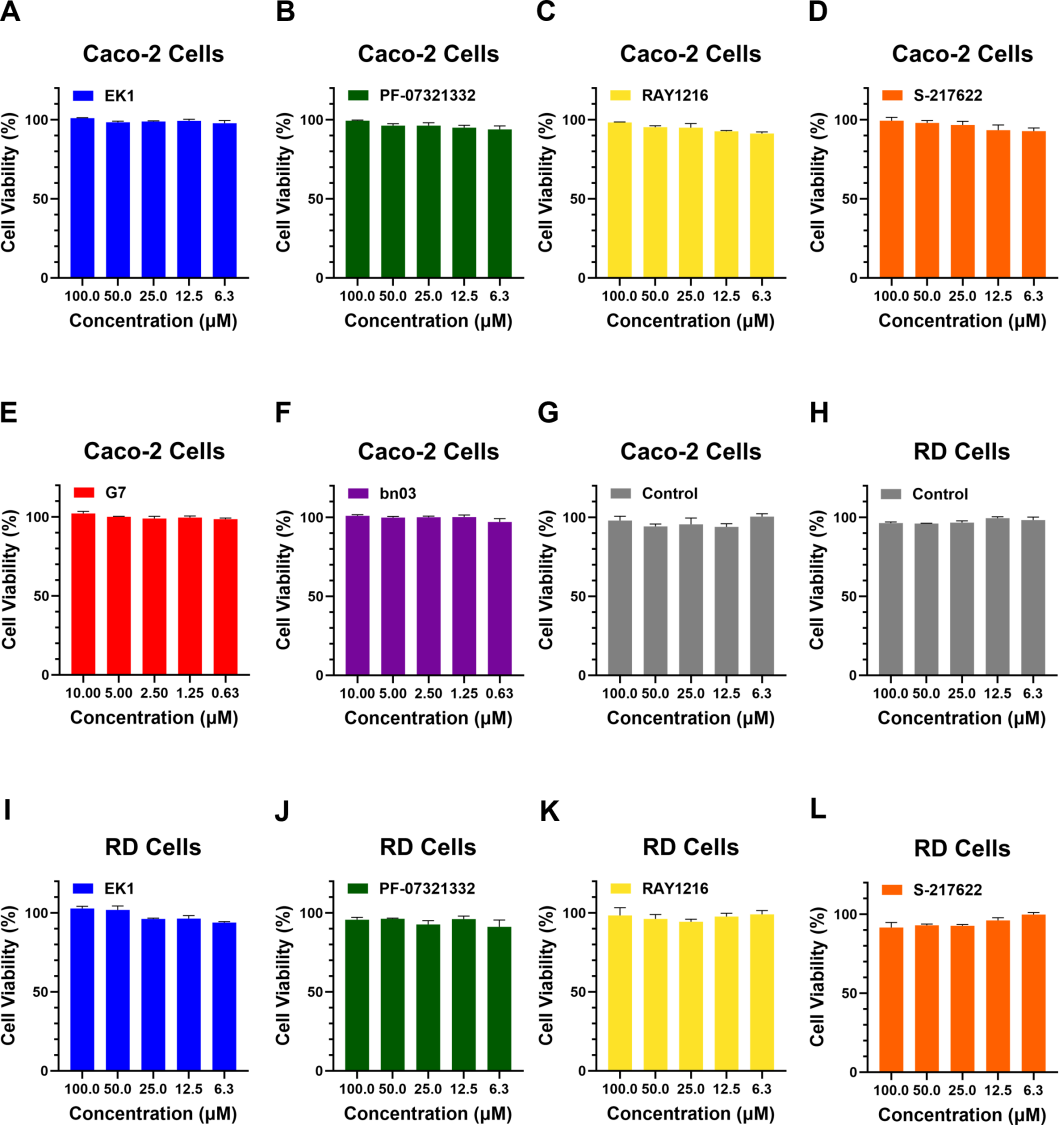
Cytotoxicity of EK1, antibodies, and M^pro^ inhibitors on Caco-2 and RD cells. (**A–G**) Cytotoxicity of EK1, PF-07321332, RAY1216, S-217622, G7, bn03, and control on Caco-2 cells. (**H–L**) Cytotoxicity of EK1, PF-07321332, RAY1216, S-217622, and control on RD cells. Data represent mean ± SD from three independent experiments (*n* = 3).

**Fig 3:**
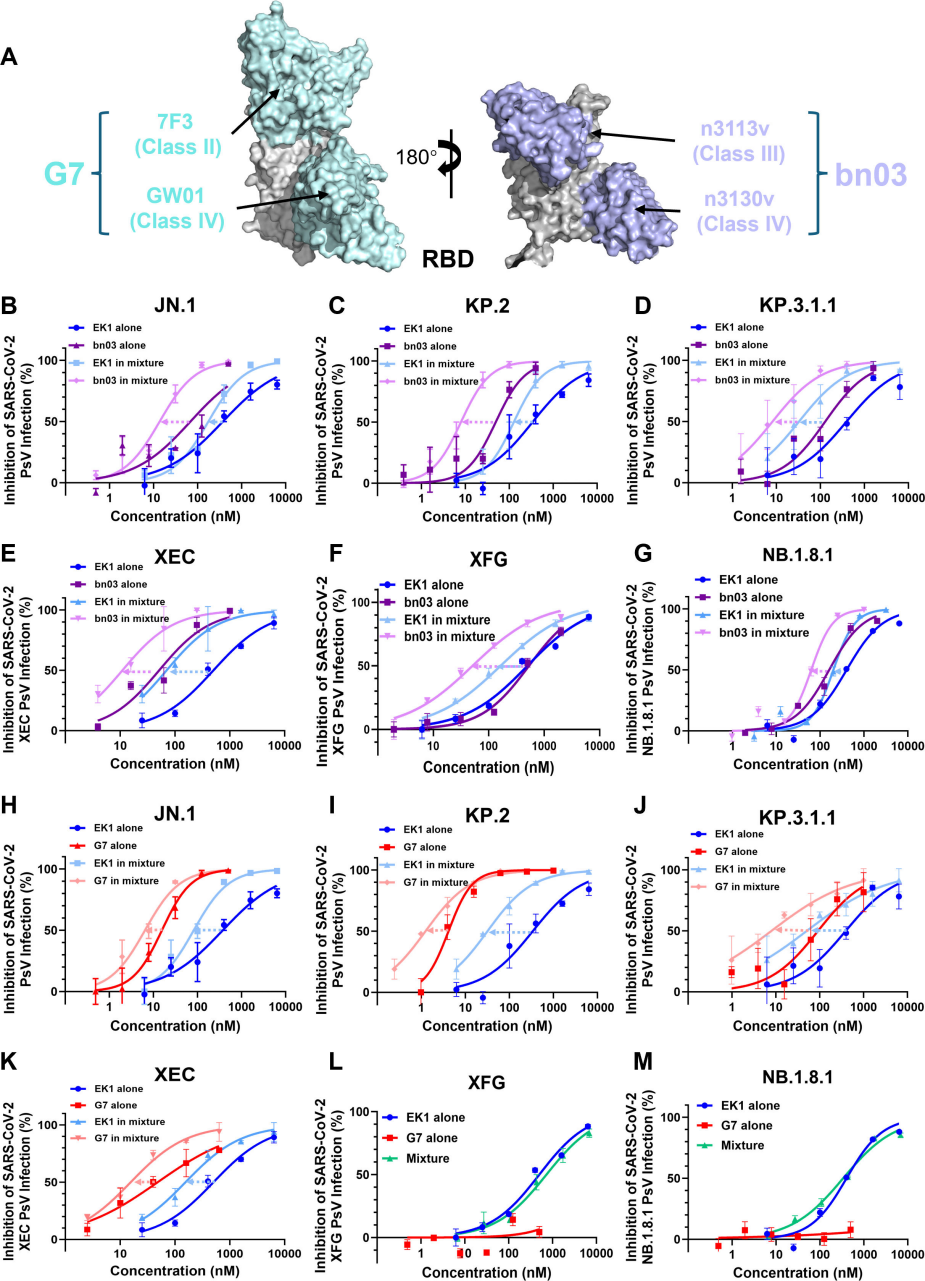
Synergistic inhibition of SARS-CoV-2 psuedovirus (PsV) infection by the EK1 peptide in combination with bnAbs. (**A**) Binding sites of bn03 (PDB ID: 7WHJ) and G7 (PDB ID: 8YWE) in the RBD of the SARS-CoV-2 S protein. (**B–G**) Dose-response curves for EK1 peptide, broadly neutralizing antibody bn03, and their combination against JN.1 PsV (**B**), KP.2 PsV (**C**), KP.3.1.1 PsV (**D**), XEC PsV (**E**), XFG PsV (**F**), and NB.1.8.1 PsV (**G**). (**H–M**) Dose-response curves for EK1 peptide, broadly neutralizing antibody G7, and their combination against JN.1 PsV (**H**), KP.2 PsV (**I**), KP.3.1.1 PsV (**J**), XEC PsV (**K**), XFG PsV (**L**), and NB.1.8.1 PsV (**M**). Combination doses were determined using the diagonal constant-ratio (fixed-ratio) method based on the IC_50_ of each agent. Data are presented as mean ± SD from three independent experiments (*n* = 3).

Based on antigenic cartography analysis and functional profiling, anti-RBD antibodies are categorized into four classes (classes 1–4) (23, 32). To evaluate combinatorial effects, we selected two bispecific antibodies: the nanobody bn03 (classes 3/4) (21), which has entered clinical trials, and the recently reported fully humanized bispecific antibody G7-Fc (classes 1/4) (Fig. 3A) (22). We first evaluated the cytotoxicity of EK1 and the antibodies bn03 and G7 on Caco-2 cells. We found that when EK1 was at a maximum concentration of 100 μM and antibodies bn03 and G7 at a maximum concentration of 10 μM, cell viability was close to 100%, indicating that EK1, bn03, and G7 are not toxic to Caco-2 cells at effective concentrations (Fig. 2A and E through G). To evaluate the synergistic antiviral efficacy of EK1 in combination with neutralizing monoclonal antibodies, we first tested three different EK1-antibody pairings against SARS-CoV-2 authentic BA.2 and Omicron pseudoviruses (PsVs), including the circulating JN.1 and KP.2 subvariants, as well as the antibody-resistant KP.3.1.1 variant. Across all tested combinations, EK1 significantly increased the neutralization potency of bnAbs, as evidenced by combination indices (CIs) ranging from 0.175 to 0.713 (Fig. 3B through D and H through J; Table 1). Among these combinations, 37.5% displayed strong synergy (CI < 0.3), while 50% exhibited moderate synergy (CI = 0.3–0.7). Notably, these synergistic effects resulted in substantial dose reductions for both components, with bnAb usage decreased 2.78- to 17.36-fold and EK1 2.82- to 15.43-fold (Table 1). The EK1/bn03 combination exhibited the most pronounced enhancement, achieving an 8.53-fold reduction in EK1 dosage and a 17.36-fold reduction in bn03 dosage against KP.3.1.1 PsV (Fig. 3D; Table 1). Furthermore, we evaluated the synergistic antiviral efficacy of EK1 combined with antibodies against pseudoviruses of the 2025 epidemic strains, including XEC, XFG, and NB.1.8.1. The combination maintained strong synergy against these emerging variants, enabling a 4.09- to 12.05-fold reduction in the required dose of neutralizing antibodies and a 2.92- to 9.90-fold reduction for EK1 (Fig. 3E through G and K through M; Table S1). Notably, while the XFG and NB.1.8.1 PsVs exhibited immune escape from the antibody G7, EK1 retained full antiviral activity, underscoring its role as a robust framework for synergistic combinations (Fig. 3L and M).

**TABLE 1:** Combination index and dose reduction of EK1 and antibody-mediated inhibition against diverse pseudotyped and authentic SARS-CoV-2

	CI	Concentration (nM)		Dose reduction	Concentration (nM)		Dose reduction
		Alone	Mix		Alone	Mix	
Pseudotyped JN.1							
			EK1			bn03	
IC_50_	0.457	467.5 ± 44.3	126.5 ± 17.1	3.70	52.9 ± 6.0	9.9 ± 1.3	5.34
			EK1			G7	
IC_50_	0.457	467.5 ± 44.3	70.1 ± 19.9	6.67	17.9 ± 5.6	5.5 ± 1.6	3.25
Pseudotyped KP.2							
			EK1			bn03	
IC_50_	0.444	470.6 ± 41.8	106.5 ± 11.9	4.42	30.5 ± 4.1	6.7 ± 0.7	4.55
			EK1			G7	
IC_50_	0.284	470.6 ± 41.8	30.5 ± 3.0	15.43	5.4 ± 1.0	1.2 ± 0.3	4.50
Pseudotyped KP.3.1.1							
			EK1			bn03	
IC_50_	0.175	341.0 ± 80.6	40.0 ± 3.3	8.53	138.9 ± 6.5	8.0 ± 0.8	17.36
			EK1			G7	
IC_50_	0.292	341.0 ± 80.6	62.7 ± 10.5	5.44	72.8 ± 7.3	7.8 ± 1.6	9.33
Authentic BA.2							
			EK1			bn03	
IC_50_	0.400	354.9 ± 25.3	119.2 ± 6.7	2.98	86.5 ± 1.8	5.6 ± 0.3	15.45
			EK1			G7	
IC_50_	0.713	354.9 ± 25.3	125.9 ± 7.1	2.82	28.1 ± 0.9	10.1 ± 0.6	2.78

### EK1 shows synergistic interactions with M^pro^ inhibitors against authentic SARS-CoV-2

**Fig 4:**
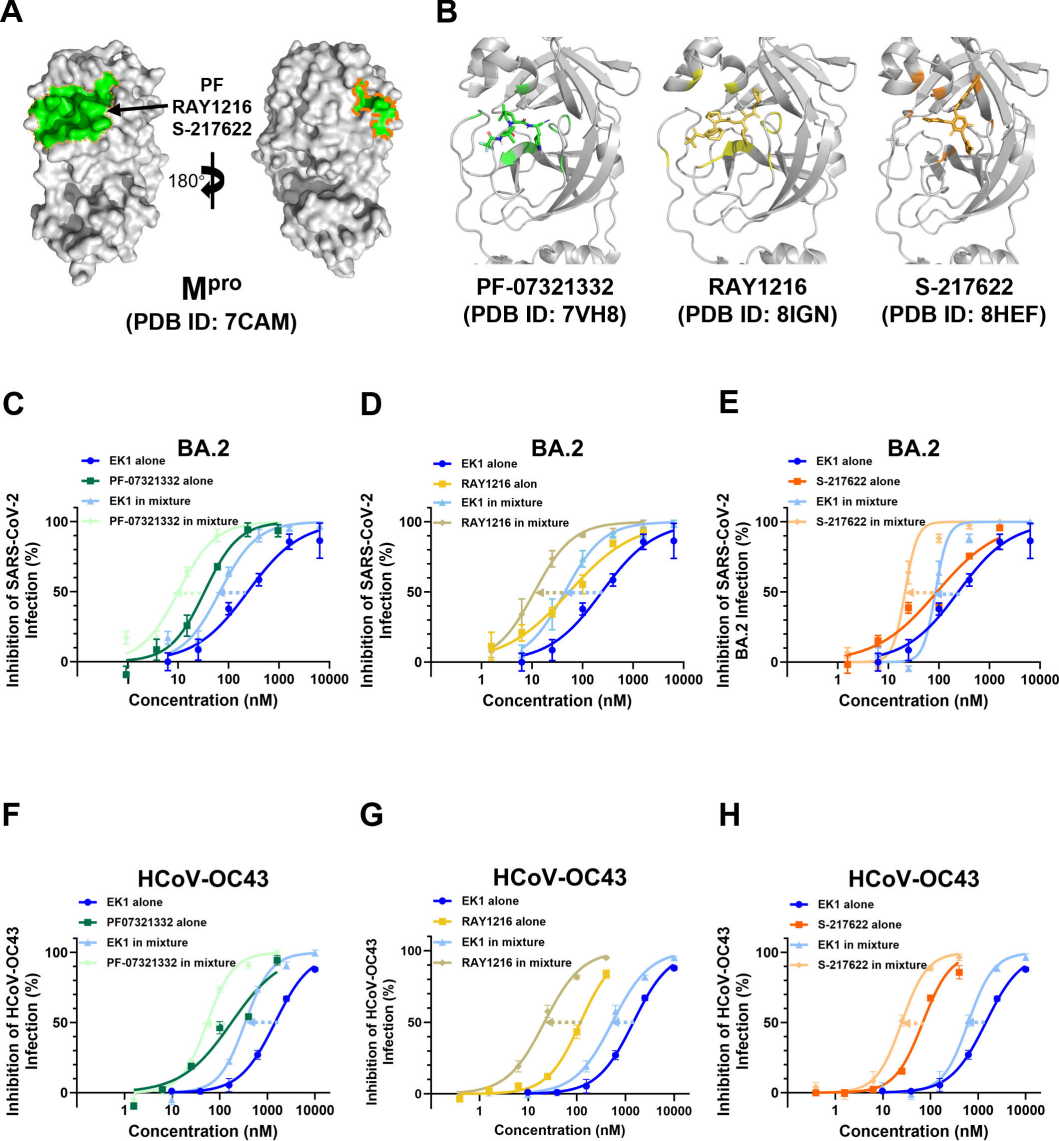
Synergistic inhibition of authentic SARS-CoV-2 BA.2 and HCoV-OC43 by dual antiviral combinations. (**A**) Crystal structure of the M^pro^ active site (PDB ID: 7CAM). The protease is shown as a semi-transparent surface in gray, with the inhibitor-binding cleft highlighted in green. (**B**) Binding sites of M^pro^ with PF-07321332 (PDB ID: 7VH8) in green, RAY1216 (PDB ID: 8IGN) in yellow, and S-217622 (PDB ID: 8HEF) in orange. (**C–H**) Dose-response analyses of the fusion inhibitor EK1 in combination with three distinct M^pro^ inhibitors against authentic SARS-CoV-2 BA.2 (**C–E**) and HCoV-OC43 (**F–H**). In each panel, EK1 and the respective M^pro^ inhibitor were tested both individually and as fixed-ratio combinations (molar ratios: PF-07321332, 20:3 for BA.2 and 25:4 for OC43; RAY1216, 4:1 for BA.2 and 25:1 for OC43; S-217622, 4:1 for BA.2 and 25:1 for OC43). Viral inhibition was quantified by fluorescent plaque assay (BA.2), and cell viability was assessed using the cell counting kit-8 (CCK-8) assay (OC43) at 48 h post-infection. Data represent mean ± SD from three independent experiments (*n* = 3).

To evaluate the robustness of our combination strategy, we selected three clinically significant M^pro^ inhibitors with distinct chemical properties: PF-07321332 (Nirmatrelvir), RAY1216 (Leritrelvir), and S-217622 (Ensitrelvir). PF-07321332 is a reversible covalent peptidomimetic inhibitor that targets the catalytic Cys145 residue of M^pro^ (33). RAY1216 is also a covalent peptidomimetic inhibitor engineered to have an improved pharmacokinetic profile and reduce dependency on ritonavir boosting (31). In contrast, S-217622 is a non-covalent, non-peptidomimetic inhibitor that occupies the substrate-binding pocket through extensive hydrogen bonding and hydrophobic networks, which is associated with a different resistance profile compared to covalent inhibitors (Fig. 4A and B) (34). Similarly, given the limited research on EK1 combined with replication inhibitors, we investigated potential synergistic interactions between these two agents. We evaluated the cytotoxicity of EK1 and M^pro^ inhibitors on Caco-2 cells. We found that when M^pro^ inhibitors (PF-07321332, RAY1216, and S-217622) were at a maximum concentration of 100 μM, cell viability was close to 100%, indicating that M^pro^ inhibitors are not toxic to Caco-2 cells at effective concentrations (Fig. 2B through D). To further assess the combinatorial potential of EK1 combined with antivirals targeting the replication stage, we tested its interaction with three M^pro^ inhibitors, PF-07321332, RAY1216, and S-217622, against authentic SARS-CoV-2 BA.2. All three combinations exhibited measurable synergy with CI values indicating significant dose-sparing effects. EK1 dosage was reduced 3.84- to 5.23-fold, while M^pro^ inhibitors exhibited consistent dose reduction of approximately 3.43- to 3.48-fold. The combination of EK1 with RAY1216 showed the strongest synergy, achieving a 5.23-fold reduction in EK1 dosage and a 3.48-fold reduction in RAY1216 dosage (Fig. 4C through E; Table 2).

**TABLE 2:** Combination index and dose reduction of EK1 and drug inhibition against authentic coronavirus

	CI	Concentration (nM)		Dose reduction	Concentration (nM)		Dose reduction
		Alone	Mix		Alone	Mix	
Authentic SARS-CoV-2 BA.2							
			EK1		PF-07321332		
IC_50_	0.552	298.9 ± 16.7	77.8 ± 3.9	3.84	27.1 ± 3.0	7.9 ± 0.6	3.43
			EK1		RAY1216		
IC_50_	0.479	298.9 ± 16.7	57.2 ± 2.4	5.23	49.8 ± 8.3	14.3 ± 0.6	3.48
			EK1		S-217622		
IC_50_	0.552	298.9 ± 16.7	77.8 ± 5.4	3.84	66.8 ± 3.7	19.5 ± 1.4	3.43
Authentic HCoV-OC43							
			EK1		PF-07321332		
IC_50_	0.712	1,618.5 ± 99.8	432.5 ± 39.0	3.74	155.5 ± 11.7	69.2 ± 6.4	2.25
			EK1		RAY1216		
IC_50_	0.750	1,618.5 ± 99.8	768.3 ± 40.3	2.11	111.4 ± 0.7	30.7 ± 3.8	3.63
			EK1				
IC_50_	0.823	1,618.5 ± 99.8	742.5 ± 20.6	2.18	81.5 ± 4.2	29.7 ± 0.8	2.74

### EK1-based combinations show broad-spectrum activity against other coronaviruses

We evaluated the cytotoxicity of EK1 and M^pro^ inhibitors on RD cells. We found that when EK1 and M^pro^ inhibitors (PF-07321332, RAY1216, and S-217622) were at a maximum concentration of 100 μM, cell viability was close to 100%, indicating that EK1 and M^pro^ inhibitors are not toxic to RD cells at effective concentrations (Fig. 2H through L). To determine whether a strong synergistic trend could extend to other human coronaviruses, we evaluated the same EK1 peptide/M^pro^ inhibitor combinations as those noted above against HCoV-OC43 infection. Consistent with the results observed for SARS-CoV-2, all combinations exhibited significant synergy with dose reductions for EK1 and M^pro^ inhibitors ranging from 2.11- to 3.74-fold and 2.25- to 3.63-fold, respectively (Fig. 4F through H; Table 2). These findings highlight the potential of EK1-based combinations as a broad-spectrum antiviral strategy.

### Triple-agent therapy produces potent and complementary antiviral effects

**Fig 5:**
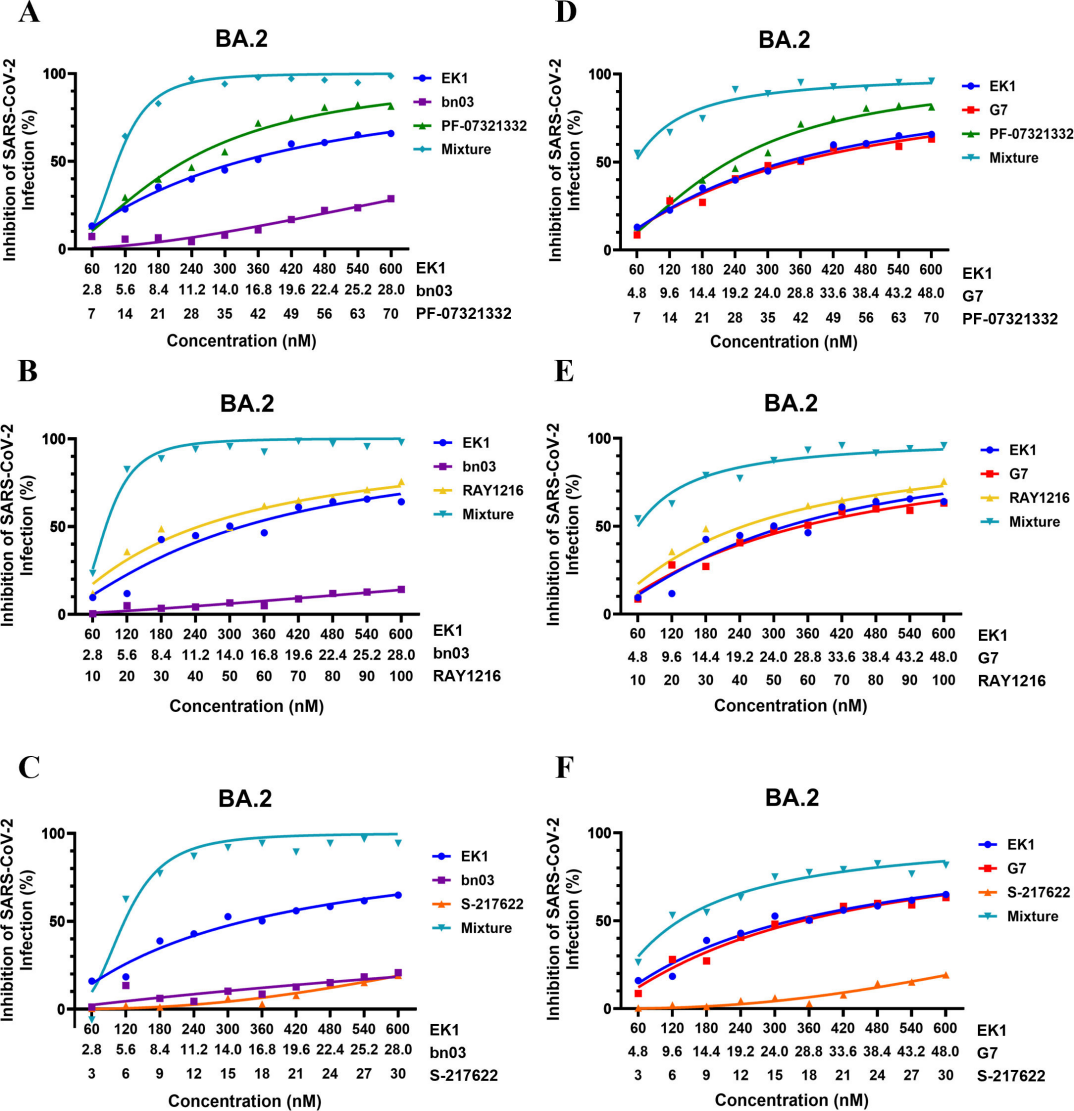
Synergistic inhibition of authentic SARS-CoV-2 BA.2 by triple antiviral combinations. (**A–F**) Dose-response analyses of the fusion inhibitor EK1 in combination with a bnAb and a replication inhibitor against authentic SARS-CoV-2 BA.2. In each panel, EK1 and the respective bnAb and replication inhibitor were tested both individually and as fixed-ratio combinations. Viral inhibition was quantified by fluorescent plaque assay (BA.2) at 48 h post-infection. Data represent mean ± SD from three independent experiments (*n* = 3).

Given the strong synergy between EK1 and bnAbs, and the broad-spectrum activity between EK1 and M^pro^ inhibitors, we further examined combinations of three agents: EK1, bnAbs, and M^pro^ inhibitors. We found that 50% of the tested triple combinations exhibited synergism (0.3 ≤ CI < 0.7) against authentic SARS-CoV-2 BA.2, while the remaining combinations showed moderate synergism (0.7 ≤ CI < 0.85) (Fig. 5; Table 3). Among these, the triple combination of EK1, G7, and PF-07321332 emerged as the most effective, allowing for dose reductions of 6.46-fold for EK1, 6.39-fold for G7, and 4.75-fold for PF-07321332, respectively (Fig. 5D; Table 3). These results underscore the potent and complementary antiviral effects achieved through rational combination therapy targeting multiple stages of the coronavirus life cycle (fusion, entry, and replication).

**TABLE 3:** Combination index and dose reduction of EK1, antibody, and drug inhibition against authentic SARS-CoV-2 BA.2

	CI	Concentration (nM)		Dose reduction	Concentration (nM)		Dose reduction	Concentration (nM)		Dose reduction
		Alone	Mix		Alone	Mix		Alone	Mix	
			EK1			bn03		PF-07321332		
IC_50_	0.768	354.9 ± 25.3	106.7 ± 4.9	3.33	86.5 ± 1.8	5.0 ± 0.2	17.30	30.4 ± 3.2	12.5 ± 0.6	2.43
			EK1			bn03		RAY1216		
IC_50_	0.532	354.9 ± 25.3	72.9 ± 4.3	4.87	86.5 ± 1.8	3.4 ± 0.2	25.44	42.2 ± 3.4	12.1 ± 0.7	3.49
			EK1			bn03		S-217622		
IC_50_	0.846	354.9 ± 25.3	211.7 ± 4.1	1.68	86.5 ± 1.8	9.9 ± 0.2	8.74	78.4 ± 5.0	10.6 ± 0.2	7.40
			EK1			G7		PF-07321332		
IC_50_	0.522	354.9 ± 25.3	54.9 ± 3.9	6.46	28.1 ± 0.9	4.4 ± 0.3	6.39	30.4 ± 3.2	6.4 ± 0.5	4.75
			EK1			G7		RAY1216		
IC_50_	0.648	354.9 ± 25.3	67.4 ± 4.1	5.27	28.1 ± 0.9	5.4 ± 0.3	5.20	42.2 ± 3.4	11.2 ± 0.7	3.77
			EK1			G7		S-217622		
IC_50_	0.840	354.9 ± 25.3	133.3 ± 8.3	2.66	28.1 ± 0.9	10.7 ± 0.7	2.63	78.4 ± 5.0	6.7 ± 0.4	11.70

Furthermore, to analyze the synergistic effects of EK1, G7, and PF-07321332 and make projections about combinatorial drug potency at concentrations that were not measured experimentally, we applied the ZIP, HSA, and Bliss models by SynergyFinder 3.0. We found that the combination of EK1, G7, and PF-07321332, either all three together or in pairs, exhibited strong synergistic inhibition of SARS-CoV-2 infection (S > 10, Fig. S1A through C). Notably, the synergy was optimal when EK1 was at 300 nM, with average synergy scores of 24.924, 34.678, and 24.352 for ZIP, HSA, and Bliss, respectively (Fig. S1D through G). At low to medium drug concentrations, the synergistic efficacy in these regions was relatively high.

## DISCUSSION

The urgent need for broad-spectrum, resistance-tolerant coronavirus therapeutics has been underscored by decades of research on viral evolution and the limitations of monotherapy. Early studies on SARS-CoV and MERS-CoV revealed that single-target interventions—whether antibody-based or small-molecule inhibitors—are susceptible to escape mutations, as evidenced by the rapid loss of efficacy of RBD-targeting antibodies against emerging SARS-CoV-2 variants (11, 18, 19, 38). Similarly, M^pro^ inhibitors such as PF-07321332 (Paxlovid) have exhibited reduced effectiveness over time due to resistance-associated mutations in the protease active site (20, 39). Our study builds on these foundational findings by designing a multi-stage combination strategy that addresses these challenges, leveraging the unique properties of the pan-coronavirus fusion inhibitor EK1.

A key insight from our work is the synergistic enhancement of bnAb potency by EK1, which aligns with and extends previous findings on fusion inhibitor-antibody combinations. Notably, EK1 has successfully completed Phase I/II clinical trials, confirming its clinical translational feasibility, where intranasal administration has been shown to be safe and effective in achieving therapeutic levels within the human respiratory tract. This further validates that the synergistic concentrations identified in our study are pharmacologically achievable in a clinical setting. Earlier studies on HIV-1 fusion inhibitors (e.g., enfuvirtide) demonstrated modest synergy with neutralizing antibodies, but these effects were limited to entry blockade (30, 40). In contrast, our data show that EK1—by targeting the conserved HR1 domain of the spike protein (37, 41–44)—induces robust, broad-spectrum synergy with diverse bnAbs across clinically relevant SARS-CoV-2 variants, including the highly antibody-resistant KP.3.1.1. The combination indices (CIs < 0.7) and dramatic dose reductions (up to 17.40-fold for bn03) observed here are particularly notable, as they address a critical clinical challenge: reducing drug dosages to minimize potential side effects while maintaining efficacy against resistant strains. This synergy likely arises from complementary mechanisms targeting distinct viral life cycle phases: bnAbs block RBD-ACE2 binding (21, 22), while EK1 disrupts the post-attachment membrane fusion step (42, 44)—a “double hit” that limits viral escape by requiring mutations in both the mutable RBD and conserved HR1 domain. Furthermore, our findings align with recent studies underscoring the necessity of developing potent multi-target cocktail therapies as the most promising approach to achieving broad-spectrum efficacy and therapeutic robustness against emerging viral variants (45).

Our findings on EK1-M^pro^ inhibitor combinations further expand the landscape of multi-stage antiviral strategies. Prior research on combination therapies for coronaviruses has primarily focused on antibody-antibody pairings (27–29), with limited exploration of fusion inhibitor-replication inhibitor combinations. We demonstrate that EK1 synergizes with three distinct M^pro^ inhibitors (PF-07321332, RAY1216, or S-217622) against authentic SARS-CoV-2 BA.2, with consistent dose-sparing effects (3.43- to 5.22-fold reductions) and *CIs* indicating significant synergy. This is particularly relevant given that M^pro^ inhibitors act intracellularly to block viral polyprotein processing (46, 47), while EK1 acts extracellularly to prevent membrane fusion—creating a spatially distinct target that reduces the likelihood of cross-resistance (48). Extending these findings to HCoV-OC43 (an Embecovirus) further validates the broad-spectrum potential of EK1-based combinations, as HCoV-OC43 shares conserved HR1 and M^pro^ domains with SARS-CoV-2 but diverges in other structural proteins (47). This cross-genus efficacy addresses a longstanding gap in coronavirus therapeutics, where most strategies are limited to Sarbecoviruses (e.g., SARS-CoV, SARS-CoV-2) (49).

**Fig 6:**
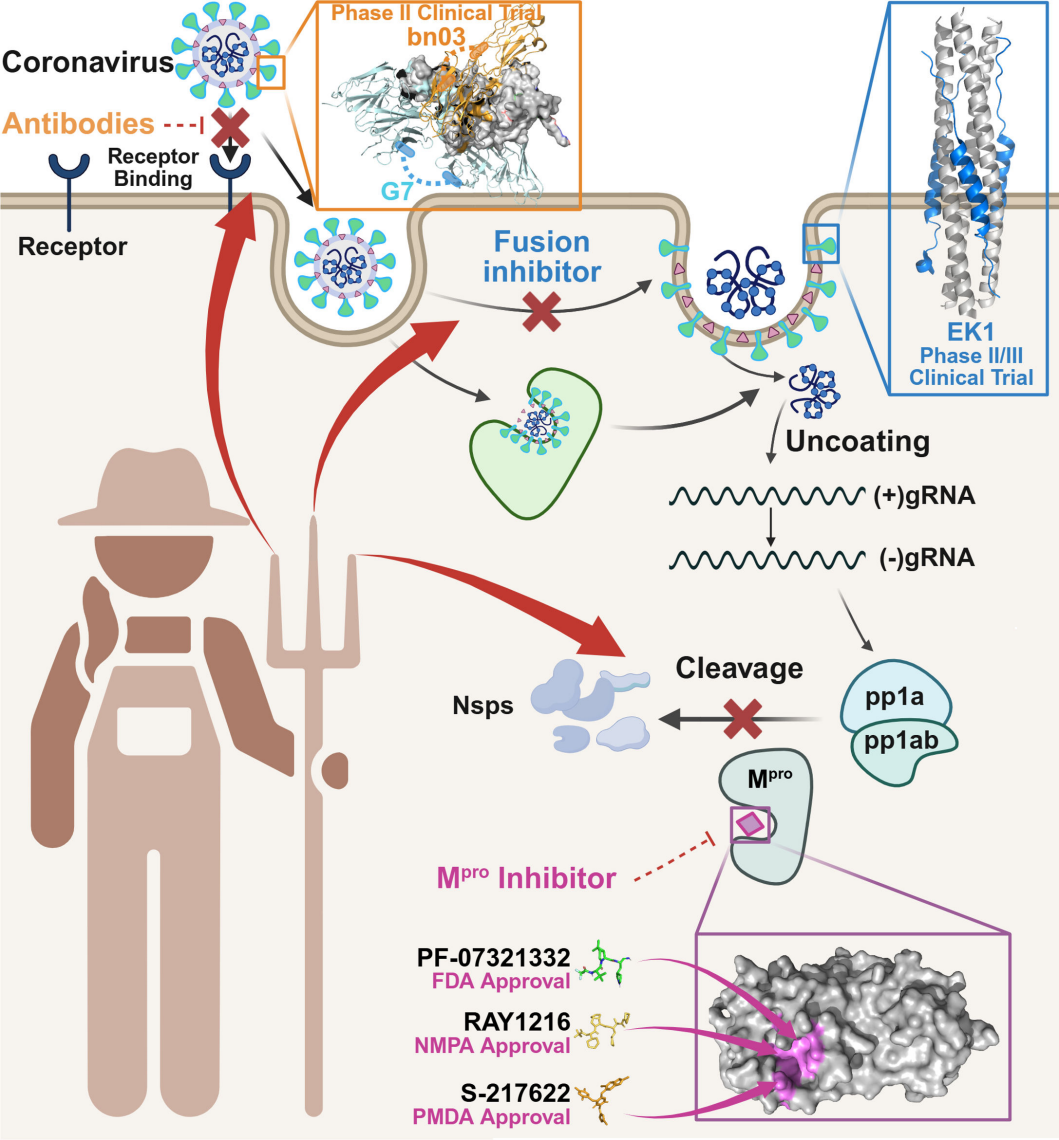
Conceptual framework for combinatorial antiviral trident therapy. Schematic overview illustrating the underlying rationale and mechanistic logic of multi-agent intervention against coronavirus infection. By simultaneously targeting discrete stages of the viral life cycle, including membrane fusion (EK1, PDB ID: 7C53), receptor engagement (neutralizing antibodies G7 (PDB ID: 8YWE) and nanobodies bn03 (PDB ID: 7WHJ) against RBD), and viral replication (M^pro^ inhibitors: PF-07321332 [PDB ID: 7VH8]; RAY1216 [PDB ID: 8IGN]; S-217622 [PDB ID: 8HEF]), this approach achieves potent, synergistic inhibition of viral entry, genome replication, and progeny virion production. Dual- or triple-agent combinations permit dose reduction of individual compounds, thereby minimizing toxicity and cost, while curtailing the emergence of resistant variants through multifaceted blockade. Trident therapy defines a modular platform for rapid deployment of broad-spectrum antivirals against SARS-CoV-2 and future zoonotic coronaviruses.

The triple combination of EK1, bnAbs, and M^pro^ inhibitors represents the most innovative aspect of our study, as it integrates three orthogonal mechanisms of action to maximize efficacy and resistance barriers (Fig. 6). Previous triple-combination studies for SARS-CoV-2 have relied on antibody cocktails plus a replication inhibitor (28), but these approaches still depend on mutable RBD targets and lack a fusion inhibition component. Our data show that adding EK1 to bnAb-M^pro^ inhibitor pairings enhances synergy (CIs 0.3–0.85) and further reduces dosages (up to 6.46-fold for EK1, 6.41-fold for G7, 4.73-fold for PF-07321332 in the top combination). The modular nature of this strategy is another key advantage: as new variants emerge, bnAbs or M^pro^ inhibitors can be swapped out without altering the EK1 backbone, enabling rapid adaptation to evolving viral threats.

The emergence of viral resistance is a pivotal challenge for antiviral therapies. Drawing on the established paradigm from HIV-1 and HCV treatment, increasing the genetic barrier to resistance is critical for sustained efficacy (50, 51). Our study utilizes a triple-combination t that concurrently attacks three independent stages of the viral life cycle: receptor binding (neutralizing antibodies), membrane fusion (EK1), and viral replication (M^pro^ inhibitors). Theoretically, the probability of a virus simultaneously acquiring escape mutations against all three independent targets is exponentially lower than for monotherapy (52). Indeed, our results with the naturally occurring variants XFG and NB.1.8.1 serve as a proof-of-concept: while these variants have evolved to escape specific neutralizing antibodies (e.g., G7), they remain susceptible to the fusion inhibitor EK1, and the combination retains potent antiviral activity. This “safety net” mechanism highlights the robustness of our multi-target strategy in mitigating the risk of resistance development.

Despite the potent synergy observed, this study has several limitations. First, our evaluations were conducted strictly *in vitro*; the complex metabolic profiles and synergistic potential *in vivo* remain to be validated in animal models. Second, unlike chronic infections such as HIV, SARS-CoV-2 causes an acute infection with a narrow therapeutic window, necessitating early intervention for a “trident therapy” to be effective. Furthermore, the clinical development of such a regimen faces challenges including potential drug-drug interactions, regulatory hurdles for multi-component drugs, and the logistical difficulty of synchronizing different delivery routes.

Taken together, our findings provide proof of concept for a rational, modular approach to coronavirus therapy that leverages the conserved fusion inhibitor EK1 as a backbone for combinations with bnAbs and M^pro^ inhibitors. This strategy addresses the key limitations of current monotherapies—variant susceptibility and resistance development—by targeting three distinct stages of the viral life cycle (Fig. 6). The design principle, which anchors combination therapy to a conserved fusion inhibitor, supports pandemic preparedness strategies against the broader coronavirus family and potentially other respiratory viruses.

## MATERIALS AND METHODS

### Materials

Human embryonic kidney 293T cells and Caco-2 cells were obtained from the American Type Culture Collection. The pan-CoV fusion inhibitor peptide EK1 was synthesized by Synpeptide (Nanjing, China). Monoclonal antibodies bn03 and G7 were kindly provided by Prof. Tianlei Ying, Prof. Jinghe Huang, and Prof. Qiao Wang (Fudan University), respectively. The small-molecule M^pro^ inhibitors nirmatrelvir (PF-07321332), RAY1216, and ensitrelvir (S-217622) were purchased from MedChemExpress (Shanghai, China).

### Collection of coronavirus spike protein sequences

Protein sequence alignment and phylogenetic analysis were performed using the MEGA12 program (38). S protein sequences of β-CoVs were employed for mutation frequency and phylogenetic analysis. Mutation landscapes were examined and visualized using GraphPad Prism 10.5.0. Key amino acid residues within the proteins were sourced from the COVID-19 CG database (https://covidcg.org).

### Generation of coronavirus pseudotyped viruses (PsVs)

Expression plasmids encoding spike proteins of SARS-CoV-2 variants were synthesized by Beijing Liuhe BGI Biotechnology and cloned into the pcDNA3.1 vector. Pseudoviruses were generated as previously described (48). Briefly, 293T cells were co-transfected with the spike-encoding plasmid and pNL4-3.Luc.RE, an HIV-1 backbone with a luciferase reporter at a 1:3 mass ratio using VigoFect transfection reagent (Vigorous Biotechnology, Beijing, China). At 8 h post-transfection, the medium was replaced with DMEM containing 10% fetal bovine serum (FBS). After incubation for 48 h at 37°C with 5% CO_2_, viral supernatants were harvested, centrifuged at 3,000 × *g* for 5 min, and filtered through 0.45 μm membranes. For infection assays, target cells were exposed to diluted pseudovirus, followed by incubation for 48 h. Luciferase activity was quantified using a luciferase assay system (Vazyme, Nanjing, China), and infectivity was measured in relative light units (RLU).

### Pseudovirus-based inhibition assay

To assess inhibitory activity against pseudotyped SARS-CoV-2, Caco-2 cells were seeded at a density of 1.2 × 10^4^ cells/well in a 96-well cell culture plate and cultured for 24 h. Test agents (Ab, EK1, or combinations) were serially diluted in a fourfold manner in serum-free DMEM and mixed in a 1:1 (vol/vol) ratio with pseudovirus suspensions. After a 40 min incubation at 37°C, the mixtures were transferred to cell monolayers. Following adsorption for 12 h, the medium was replaced with DMEM supplemented with 10% FBS and incubated for 48 h. Luciferase activity was measured, and inhibition rates were calculated as follows: inhibition (%) = [1 − (RLU_sample − RLU_blank) / (RLU_control − RLU_blank)] × 100.

Half-maximal inhibitory concentrations (IC_50_) were determined using a four-parameter logistic regression model (GraphPad Prism 10.5.0).

### Fluorescent focus reduction assay

To assess the inhibitory activity of agents (EK1, replication inhibitors, or combinations) against authentic SARS-CoV-2 BA.2 virus, Caco-2 cells were seeded at a density of 1.2 × 10^4^ cells/well in a 96-well plate for 24 h. Test agents (EK1, replication inhibitors, or combinations) were serially diluted fourfold in serum-free DMEM and mixed in a 1:1 (vol/vol) ratio with authentic SARS-CoV-2 BA.2 virus (0.005 MOI). Another antiviral agent (EK1, Ab, replication inhibitors, or combinations), starting from 1 × IC_50_, was prepared to cover nine descending concentrations (90% to 10% of the initial dose) and four descending concentrations (serially diluted twofold). After 30 min of incubation at 37°C, mixtures were added to the cell monolayers. After adsorption for 2 h, cells were overlaid with 1% sodium carboxymethyl cellulose (Sigma, Shanghai, China) in serum-free DMEM and incubated for 48 h.

Cells were fixed with 4% paraformaldehyde (Beyotime, Shanghai, China) for 30 min, blocked with 3% bovine serum albumin (Solarbio, Beijing, China) for 1 h, stained with an anti-nucleocapsid protein mouse polyclonal antibody (Sino Biological, Beijing, China) at 4°C overnight, and stained with Alexa Fluor 488-conjugated goat anti-mouse antibody (Thermo, Shanghai, China) for 1 h at room temperature in the dark. Fluorescence signals were captured using a high-content imaging system (Tecan Spark Cyto, Männedorf, Switzerland).

### HCoV-OC43 replication inhibition assay

To evaluate antiviral activity against HCoV-OC43, RD cells were seeded at 1.2 × 10^4^ cells/well in a 96-well plate for 24 h. Serial fourfold dilutions of the agents (EK1, M^pro^ inhibitors, or combinations) were mixed in a 1:1 (vol/vol) ratio with virus in serum-free DMEM. Following a 30 min incubation at 37°C, mixtures were added to the RD cell monolayers. At 8 h post-infection, the medium was replaced with DMEM supplemented with 2% FBS, and cells were incubated for 48 h. Cytopathic effects were quantified using the CCK-8 assay (Dojindo, Kumamoto, Japan), in which 100 μL of a 20-fold diluted reagent was added to each well and incubated for 4 h at 37°C. Absorbance at 450 nm was measured to assess cell viability.

### Cytotoxicity assay

To evaluate cytotoxicity, Caco-2 cells and RD cells were seeded at 1.2 × 10^4^ cells/well in a 96-well plate for 24 h. The agents (EK1, antibodies, or Mpro inhibitors) were serially twofold diluted in serum-free DMEM and then added to the cell monolayers. At 12 h post-infection, the medium was replaced with DMEM supplemented with 10% FBS, and cells were incubated for 48 h. Cytopathic effects were quantified using the CCK-8 assay. Absorbance at 450 nm was measured to assess cell viability.

### Combinatorial data analysis

Synergistic effects were assessed by Chou-Talalay’s median-effect method in CompuSyn to calculate CI. CI values were interpreted as follows: CI < 0.1: very strong synergism; 0.1 ≤ CI < 0.3: strong synergism; 0.3 ≤ CI < 0.7: synergism; 0.7 ≤ CI < 0.85: moderate synergism; 0.85 ≤ CI < 0.90: slight synergism (39). Triple-agent combinations were further evaluated using three-dimensional response surface modeling. ZIP, HSA, and Bliss synergy scores of the triple-agent combinations were calculated in SynergyFinder 3.0 (53). A synergistic effect is indicated when the ZIP, HSA, and Bliss combination scores are greater than 10. All results are presented as the mean ± SD of triplicate experiments.

## Data Availability

Expression plasmids encoding spike proteins of SARS-CoV-2 variants, including JN.1 (GenBank: PP151451.1), KP.2 (GenBank: PP905415.1), KP.3.1.1 (GenBank: PV653689.1), XEC (GenBank: PV598302.1), XFG (GenBank: PV865212.1), NB.1.8.1 (GenBank: OZ277114.1), are available in the NCBI Nucleotide database (https://www.ncbi.nlm.nih.gov/nuccore). All relevant data supporting the findings of this study are included in the paper and its supplemental material. Additional data sets generated and analyzed during the present study are available from the corresponding author upon reasonable request.

## SUPPLEMENTAL MATERIAL

Supplemental material jvi.00076-26-s0001.docx Figure S1 and Table S1.

ASM does not own the copyrights to Supplemental Material that may be linked to, or accessed through, an article. The authors have granted ASM a non-exclusive, world-wide license to publish the Supplemental Material files. Please contact the corresponding author directly for reuse.
