# Supplementary material for: Potent in vitro synergistic antiviral effects of the pan-coronavirus fusion inhibitor EK1 in combination with RBD-specific antibodies or Mpro inhibitors
Source: J Virol. 2026 Mar 30;100(4):e00076-26. doi: 10.1128/jvi.00076-26 (PMC13098233; doi:10.1128/jvi.00076-26)
Supplement: Supplemental material — Figure S1 and Table S1. [file jvi.00076-26-s0001.docx]

**Potent Synergistic Antiviral Effects of the Pan-Coronavirus Fusion Inhibitor EK1 in Combination with RBD-Specific Antibodies or M^pro^ Inhibitors** **in vitro**

Ruixue Xiu^1^, Yuanzhou Wang^1^, Wenbo Cai^2^, Qian Wang^1^, Minxiang Xie^1^, Yingdan Wang^1^, Cheng Li^1^, Qiao Wang^1^, Jinghe Huang^1^, Tianlei Ying^1^, Chuanjun Song^2^, Lu Lu^1*^, Shibo Jiang^1*^, Wei Xu^1*^

^1^ Key Laboratory of Medical Molecular Virology (MOE/NHC/CAMS), Shanghai Institute of Infectious Disease and Biosecurity, Shanghai Frontiers Science Center of Pathogenic Microorganisms and Infection, School of Basic Medical Sciences, Shanghai Public Health Clinical Center, Shanghai Medical College, Fudan University, Shanghai, China

^2^ College of Chemistry, Pingyuan Laboratory, Zhengzhou University, Zhengzhou, Henan Province 450001, China

*Corresponding authors

E-mail: [shibojiang@fudan.edu.cn](mailto:shibojiang@fudan.edu.cn); [lul@fudan.edu.cn](mailto:lul@fudan.edu.cn); xuwei11@fudan.edu.cn

**Running Head:** Trident therapy against coronavirus

**Supplemental Materials**

**Figure S1.** **EK1, G7 and PF-07321332 synergize to suppress SARS-CoV-2 BA.2 infection.** (A-C) Zip, HSA and Bliss synergy scores of the three-drug and two-drug combinations as calculated in SynergyFinder 3.0. The dotted vertical line indicates the cutoff for strong synergy defined previously(27, 53). (D) The synergistic inhibitory activity (%) of EK1, G7 and PF-07321332 when the concentration of EK1 is 300 nM. (E-G) the two-dimensional topograph that highlights the areas of synergy across the full dose response matrix (EK1=300 nM). Data represent mean±SD from three independent experiments (n=3).

| **Table S1. Combination index and dose reduction of EK1 and antibody-mediated inhibition against diverse pseudotyped SARS-CoV-2** | | | | | | | |
| --- | --- | --- | --- | --- | --- | --- | --- |
|  | **CI** | **Concentration**  **(nM)** | | **Dose reduction** | **Concentration**  **(nM)** | | **Dose reduction** |
|  |  | **Alone** | **Mix** |  | **Alone** | **Mix** |  |
| **Pseudotyped XEC** | | | |  |  |  |  |
|  |  |  | **EK1** |  |  | **bn03** |  |
| **IC_50_** | 0.309 | 551.2±25.3 | 55.7±2.2 | 9.90 | 44.3±4.7 | 8.7±0.4 | 5.09 |
|  |  |  | **EK1** |  |  | **G7** |  |
| **IC_50_** | 0.572 | 551.2±25.3 | 148.7±9.3 | 3.71 | 61.0±3.7 | 14.9±0.9 | 4.09 |
| **Pseudotyped XFG** | | | |  |  |  |  |
|  |  |  | **EK1** |  |  | **bn03** |  |
| **IC_50_** | 0.382 | 530.7±18.9 | 157.2±6.6 | 3.38 | 593.1±20.5 | 49.2±2.1 | 12.05 |
| **Pseudotyped NB.1.8.1** | | | |  |  |  |  |
|  |  |  | **EK1** |  |  | **bn03** |  |
| **IC_50_** | 0.557 | 343.2±6.8 | 117.6±7.0 | 2.92 | 166.9±4.3 | 36.7±2.2 | 4.55 |
